# Evaluation of Training to Increase Knowledge of the Addendum Guidelines for the Prevention of Peanut Allergy in the US

**DOI:** 10.1001/jamanetworkopen.2023.4706

**Published:** 2023-03-24

**Authors:** Waheeda Samady, Lucy A. Bilaver, Jialing Jiang, Anahita Iyer, Joy Laurienzo Panza, Alkis Togias, Ruchi S. Gupta

**Affiliations:** 1 Ann & Robert H. Lurie Children’s Hospital of Chicago, Chicago, Illinois; 2 Northwestern University Feinberg School of Medicine, Chicago, Illinois; 3 National Institute of Allergy and Infectious Diseases, Bethesda, Maryland

## Abstract

This survey study examines changes in pediatric clinicians’ knowledge of eczema identification and the 2017 Addendum Guidelines for the Prevention of Peanut Allergy after an educational intervention.

## Introduction

Peanut allergy (PA) affects 2.2% of US children.^1^ The 2017 Addendum Guidelines for the Prevention of Peanut Allergy recommend high-risk infants (severe eczema and/or egg allergy) be evaluated for peanut sensitization before early introduction of peanut and be referred to an allergist depending on results. Infants with mild-to-moderate eczema should be introduced to peanut around 6 months of age.^2^

Successful guideline implementation falls on pediatric clinicians during 4- and 6-month well-child visits. However, previous studies reported high training needs,^3^ low implementation rates,^3^ and low knowledge of the guidelines^4^ among clinicians. This study aims to measure changes in pediatric clinicians’ guideline knowledge and eczema identification after an educational intervention.

## Methods

This survey study was approved by the Ann & Robert H. Lurie Children’s Hospital of Chicago institutional review board. Participants provided implicit consent by completing the survey. This study followed the American Association for Public Opinion Research (AAPOR) reporting guideline.

The Intervention to Reduce Early (Peanut) Allergy in Children (iREACH) study is a 2-group, cluster randomized clinical trial aiming to increase pediatric clinician adherence to guidelines. Experts developed a training video with content on PA prevention, guidelines, iREACH interventional tools, and eczema categorization. This 21.5-minute video was offered to 16 intervention practice sites in Illinois, including urban clinics, suburban clinics, rural clinics, private practices, and Federally Qualified Health Centers (FQHC). An online 34-item pretraining survey and 7-item posttraining survey were administered to all pediatric clinicians at the intervention practice sites from November 4, 2020, to January 27, 2022. Survey questions included: demographics, guideline usage, awareness, knowledge, and eczema identification. Race (American Indian or Alaskan Native, Asian, Black or African American, Native Hawaiian or Pacific Islander, White, and other) and ethnicity (Hispanic/Latino and non-Hispanic/Latino) were self-reported.

The McNemar test was used to measure proportional differences in pediatric clinicians’ knowledge pre- and post-training. Significance threshold was 2-sided *P* < .05 and testing was 2-sided. All statistical analyses were performed using SAS version 5 (SAS Institute) from December 2021 to September 2022.

## Results

Overall, 185 clinicians participated (100% completion rate). Among clinicians, 29 (15.7%) were Asian, 5 (2.7%) were Black, 20 (10.8%) were Hispanic or Latino, 134 (72.4%) were White, and 155 (83.8%) were female; 96 (51.9%) had been in clinical practice for 0 to 10 years, 50 (27.0%) for 11 to 20 years, and 23 (12.4%) for 21 to 30 years; 94 (50.8%) practiced at an FQHC.

Most pediatric clinicians (89.7% [166 of 185]) reported previous awareness. Among those aware of the guidelines, 40 (21.6%) reported full implementation and 127 (68.7%) reported no prior training (Table 1).

**Table 1.** Guideline Implementation, Knowledge, and Training Prior to the Intervention to Reduce Early (Peanut) Allergy in Children Training

Questions and answers	Clinicians, No. (%) (N = 185)
Prior to this survey, were you aware of the Guidelines for peanut allergy prevention that recommend the early introduction of peanut-containing foods into the diets of infants to prevent peanut allergy? (n = 185)	
Yes	166 (89.7)
No	19 (10.3)
Which statement best describes your use of the Guidelines for peanut allergy prevention in your practice? (n = 166)	
Not implementing	19 (10.3)
Partially implementing	106 (57.3
Fully implementing	40 (21.6)
Have you received any training around the Guidelines for peanut allergy prevention? (n = 166)	
Yes	38 (20.5)
No	127 (68.7)
Do you believe you need more education or training on the Guidelines for peanut allergy prevention? (n = 38)	
Yes	30 (16.2)
No	8 (4.3)
Which of the following items have been a barrier or concern for you in using the Guidelines for peanut allergy prevention? (Select all that apply.) (Among partial or full guideline implementation)	
Access to allergists for referrals	14 (7.6)
Insufficient insurance coverage or reimbursement	3 (1.6)
Lack of clinic time	54 (29.2)
Conducting an in-office supervised feeding of peanut	34 (18.4)
Indicate your level of agreement with the following statement: The early introduction of peanut-containing foods is an effective method for the prevention of peanut allergy (n = 185)	
Strongly agree	112 (60.5)
Agree	69 (37.3)
Neither agree nor disagree	4 (2.2)
Disagree	0 (0.0)
Strongly disagree	0 (0.0)

After training, the percentage of participants correctly answering all knowledge questions improved from 72.6% [119 of 164] to 94.5% [155 of 165] (*P* < .001) (Table 2). Questions using visual eczema vignettes had the most improvement: severe eczema identification and guideline application improved from 63.4% (104 of 165) to 97.6% (160 of 165) correct (*P* < .001) and moderate eczema improved from 53.1% (87 of 164) to 78.1% (128 of 164) correct (*P* < .001). The percentage answering all guideline application questions correctly improved from 29.0% (47 of 162) to 70.4% (114 of 162) post training (*P* < .001).

**Table 2.** Pre– and Post–Intervention to Reduce Early (Peanut) Allergy in Children Training Responses for Guidelines Knowledge Questions^a^

Questions and answers	Clinicians, No. (%)		*P* value^b^
	Pretraining survey	Posttraining survey	
For an infant aged 6 mos who does NOT have eczema or any food allergies, what would you typically do next with respect to peanut allergy prevention? (n = 165)			
Order a peanut-specific IgE/RAST test	1 (0.6)	0	NA^c^
Refer to an allergist for consultation and testing	0	0	
Recommend avoidance of peanut-containing foods	0	0	
Offer an in-office feeding of a peanut-containing food	0	0	
Recommend the introduction of peanut-containing food, in accordance with family preferences and cultural practices^c^	159 (96.4)	165 (100.0)	
I would not take any additional steps with respect to peanut allergy prevention	4 (2.4)	0	
Other	1 (0.6)	0	
For an infant aged 6 mos who has mild-to-moderate eczema, what do the Guidelines recommend next with respect to peanut allergy prevention? (Select only one.) (n = 164)			
Order a peanut-specific IgE/RAST test	7 (4.3)	7 (4.3)	<.001
Refer to an allergist for consultation and testing	11 (6.7)	0	
Recommend avoidance of peanut-containing foods	3 (1.8)	0	
Offer an in-office feeding of a peanut-containing food	4 (2.4)	0	
Recommend the introduction of peanut containing foods^c^	132 (80.5)	156 (95.1)	
I would not take any additional steps with respect to peanut allergy prevention	6 (3.7)	1 (0.6)	
Other	1 (0.6)	0	
For an infant aged 6 mos who has severe eczema and/or egg allergy, what do the Guidelines recommend next with respect to peanut allergy prevention? (Select all) (n = 165)			
Order a peanut-specific IgE test^c^	55 (33.3)	160 (97.0)	.002
Conduct peanut-specific skin prick testing in my office	1 (0.6)	3 (1.8)	
Recommend the introduction of peanut containing foods	7 (4.2)	0	
Refer to an allergist for consultation and testing^c^	126 (76.4)	93 (56.4)	
Recommend avoidance of peanut-containing foods	20 (12.1)	11 (6.7)	
Offer an in-office feeding of a peanut-containing food	3 (1.8)	3 (1.8)	
Recommend the introduction of peanut-containing food	11 (6.7)	3 (1.8	
I would not take any additional steps with respect to peanut allergy prevention	1 (0.6)	0	
Other	1 (0.6)	0	
Knowledge of Guidelines Question Summary (n = 164)			
3 correct answers	119 (72.6)	155 (94.5)	<.001
Eczema identification and guideline application questions			
A four-month-old infant presents with dry itchy skin lesions on the face and upper extremities. You quickly recognize this as atopic dermatitis. The lesions are erythematous (dull red) and indurated, and you note lichenification. There is also some crusting. The lesions are primarily on the face and arms. How would you rate the severity of the atopic dermatitis? (n = 165)			
Atopic dermatitis			
Mild	9 (5.5)	9 (5.5)	.03
Moderate^c^	139 (84.2)	150 (90.9)	
Severe	17 (10.3)	6 (3.6)	
A two-month-old African American infant presents with deep purple skin lesions on their face, scalp, extremities, and trunk. The lesions are pruritic, with lichenification, induration, oozing, and crusting. What is your diagnosis? (n = 163)			
Bruising, secondary to a bleeding disorder	1 (0.6)	0	.02
Atopic dermatitis			
Moderate	14 (8.6)	5 (3.1)	
Severe^c^	148 (90.8)	158 (96.9)	
[After viewing pictures of infant with severe eczema] In following the NIAID peanut prevention guidelines, what is the appropriate next step? (n = 165)			
Obtain a peanut sIgE/RAST^c^	104 (63.4)	160 (97.6)	<.001
Start peanut containing products in the infant’s diet immediately	10 (6.1)	3 (1.8)	
Advise the family to wait until the child is at least 9 mos old before starting peanut containing products	1 (0.6)	1 (0.6)	
Diagnose patient with a peanut allergy	0	0	
I don’t know	49 (29.9)	0	
A4. [After viewing pictures of infant with moderate eczema] When should this infant's parent add peanut containing foods to the baby's diet? (n = 164)			
Around 6 months of age^c^	87 (53.1)	128 (78.1)	<.001
They should wait until after 11 mos of age	0	0	
Only after they have been tested and cleared by an Allergist	18 (11.0)	7 (4.3)	
Peanut-containing foods can be introduced based on family preferences and cultural practices	38 (23.2)	29 (17.7)	
I don't know	21 (12.8)		
Eczema Identification and Application Question Summary (n = 162)			
4 correct answers	47 (29.0)	114 (70.4)	<.001

Abbreviations: NA, not applicable; NIAID, National Institute of Allergy and Infectious Diseases; RAST, radioallergosorbent test.

^a^ Pre- and postresponse frequencies are only presented for respondents who answered the question in both surveys.

^b^ *P* values are from the McNemar test of the null hypothesis of no treatment effect.

^c^ There was no variation in responses pre- and posttraining as 100% of clinicians answered correctly at posttraining. Therefore, a *P* value could not be calculated.

## Discussion

The iREACH training modules were developed to meet the educational needs of pediatric clinicians as they implement the guidelines. This study demonstrates how training can significantly improve guideline knowledge and application among a large group of clinicians serving diverse populations. This is consistent with past research which demonstrated that awareness among pediatricians did not reflect appropriate guidelines implementation.^5,6^ Baseline knowledge did not differ based on years of practice, highlighting the need for training across the spectrum of pediatric clinicians. The robust posttraining improvement for high-risk infants is notable as these infants require early identification to facilitate testing and/or referral. Furthermore, using standardized visual tools were effective to improving guideline application knowledge. A limitation of this study was that long-term knowledge retention was not assessed.

This study found that iREACH training is an effective and accessible method to improving guideline implementation while facilitating PA prevention. This training can also be easily updated to reflect emerging evidence on prevention of other common food allergies.

## Notes

Supplement.
Data Sharing Statement
