# Supplementary material for: Evaluation of Training to Increase Knowledge of the Addendum Guidelines for the Prevention of Peanut Allergy in the US
Source: JAMA Netw Open. 2023 Mar 24;6(3):e234706. doi: 10.1001/jamanetworkopen.2023.4706 (PMC10313145; doi:10.1001/jamanetworkopen.2023.4706)
Supplement: Supplement. — Data Sharing Statement [file jamanetwopen-e234706-s001.pdf]

Data Sharing Statement

Samady. Evaluation of Training to Increase Knowledge of the Addendum Guidelines for the Prevention of Peanut Allergy in the US. *JAMA Netw Open*. Published March 24, 2023. doi:10.1001/jamanetworkopen.2023.4706

Data
Data available: No
